# Inhibition of long non-coding RNA HOXA11-AS against neuroinflammation in Parkinson's disease model via targeting miR-124-3p mediated FSTL1/NF-κB axis

**DOI:** 10.18632/aging.202837

**Published:** 2021-04-04

**Authors:** Hua Cao, Xinsheng Han, Yonglin Jia, Baohua Zhang

**Affiliations:** 1 Department of Neurology, Kaifeng Central Hospital, Kaifeng 475000, Henan, China

**Keywords:** HOXA11-AS, Parkinson's disease, miR-124-3p, FSTL1, NF-κB

## Abstract

Background: Studies have revealed that lncRNA HOXA11-AS contributes to regulating inflammation, while the role of HOXA11-AS in Parkinson’s disease (PD) remains unclear.

Methods: Both *in vivo* and *in vitro* PD models were induced. Gain- or loss-assays of HOXA11-AS and miR-124-3p were conducted. The neurological functions, dopaminergic neurons damage, microglia activation of PD mice were measured. Afterwards, the expressions of inflammatory factors were examined with RT-PCR. Western blot was employed to detect the level of FSTL1, NF-κB and NLRP3 inflammasome. Meanwhile, bioinformatics analysis and dual-luciferase reporter assay were utilized to confirm the targeting relationships among miR-124-3p, HOXA11-AS and FSTL1.

Results: HOXA11-AS promoted MPTP-mediated SH-SY5Y neuronal injury and LPS-induced microglia activation, while miR-124-3p had the opposite effects. Additionally, miR-124-3p was the target of HOXA11-AS and FSTL1. HOXA11-AS overexpression enhanced the expression of inflammatory factors and FSTL1, NF-κB and NLRP3 inflammasome, while inhibiting NF-κB weakened HOXA11-AS-mediated neuronal damage and microglia activation. Moreover, HOXA11-AS1 downregulation ameliorated MPTP-induced neurological damages and neuroinflammation in mice.

Conclusion: Inhibition of HOXA11-AS protects mice against PD through repressing neuroinflammation and neuronal apoptosis through miR-124-3p-FSTL1-NF-κB axis.

## INTRODUCTION

PD, also known as tremor paralysis, is the second most common progressive neurodegenerative disease after Alzheimer's disease (AD), which mainly occurs in middle-aged and elderly people. PD presents with various non-motor symptoms (NMS) (such as cognitive impairment) that may occur before motor symptoms, in the early stage, or throughout the course of the disease [1, 2]. Currently, PD is mainly treated with symptomatic therapy and dopamine precursor levodopa (L-DOPA), while the dyskinesia associated with the L-DOPA treatment often influences the life quality of patients with advanced PD [3, 4]. Therefore, it is urgent to explore pathogenesis and find new treatment strategies of PD.

It’s reported that neuroinflammation is a sophisticated biological process that contributes to age-related cerebrovascular and neurodegenerative diseases, such as cerebral ischemia, AD and PD [5, 6]. As the main immune cells of the central nervous system (CNS), microglia are getting increasing attention. Studies have revealed that excessive activation of microglia and subsequent release of pro-inflammatory cytokines such as IL-1β, IL-6, TNF-α, IL-18, etc. exert key roles in PD ’s development [7].

Long non-coding RNAs (lncRNAs) belong to a class of non-coding RNAs that contain over 200 nucleotides. Recent studies have reported that lncRNAs also exert critical functions in regulating the gene expression of neuron cell differentiation, synaptogenesis, and synaptic plasticity in CNS [8]. LncRNAs could lead to expression changes of target genes, thereby contributing to the pathogenesis of neurodegenerative diseases such as PD [9]. For example, studies have shown that lncRNA-UCA1 aggravates PD development by upregulating α-synuclein (SNCA) [10]. Additionally, Lin Q et al. found that knocking down lncRNA HOTAIR, as a competitive endogenous, could inhibit PD progression, via regulation miR-126-5p/RAB3IP axis [11]. LncRNA HOXA11-AS is also an essential type of lncRNA. Previous studies have suggested that HOXA11-AS not only participates in the development of cancers [12], but also regulates inflammation [13, 14], while the potential functions of HOXA11-AS in PD, especially in microglia mediated neuroinflammation remain elusive.

MicroRNAs (miRs) also belong to the class of non-coding RNAs, with a length of only 18 to 25 nucleotides. The dysregulation of miRs was reported to correlate with the PD pathogenesis. MiR-150 expression is decreased in the serum of PD patients and could be as a candidate diagnostic biomarker for PD. Overexpressing miR-150 repressed lipopolysaccharide (LPS) mediated microglial inflammation [15]. Cai LJ et al. found that up-regulating miR-375 alleviated the damage of dopaminergic neurons, and reduced oxidative stress and inflammation by inhibiting SP1 in 6-hydroxydopamine induced PD animal model [16]. As an important member of miR, miR-124-3p was found to exert a neuroprotective role in spinal cord ischemia-reperfusion injury (SCIRI) [17] and traumatic brain injury (TBI) [18], while its role in PD needs further exploration.

Follistatin-like 1 (FSTL1) is a TGF-β superfamily binding protein that is proved to regulate the pathological process of multiple diseases [19]. Recent studies have shown that FSTL1 facilitates the inflammatory responses and cartilage degradation by activating NF-kappa B(NF-κB) signaling pathways in osteonecrosis of the femoral head (ONFH) model [20]. Meanwhile, knockdown FSTL1 attenuates the TLR4/MyD88/NF-κB and MAPK pathway, decreasing oxLDL-induced inflammation reactions in human coronary artery endothelial cells (HCAECs) [21]. Moreover, FSTL1 is regulated by transmembrane protein 3 (Ifitm3) in astrocytes, which could be involved in polyI:C-induced neurodevelopmental impairment [22], and the downregualtion of FSTL1 by miR-29a led to enhancement of neurite outgrowth of rat neural stem cells [23], suggesting that FSTL1 can also modulate CNS disease.

Presently, both *in vivo* and *in vitro* PD models were induced by 1-methyl-4-phenyl-1,2,3,6-tetrahydropyridine (MPTP) in mice and SH-SY5Y cells or by lipopolysaccharide (LPS) in BV-2 microglia cells. Then we conducted experiments to explore the biofunctions of HOXA11-AS, miR-124-3p, and FSTL1 in the development of PD. Our data suggested a novel network of HOXA11-AS and miR-124-3p-FSTL1-NF-κB axis in PD, which provided new references for clinical research and treatment of PD.

## RESULTS

### Expression characteristics of HOXA11-AS and miR-124-3p in PD

**Figure 1:**
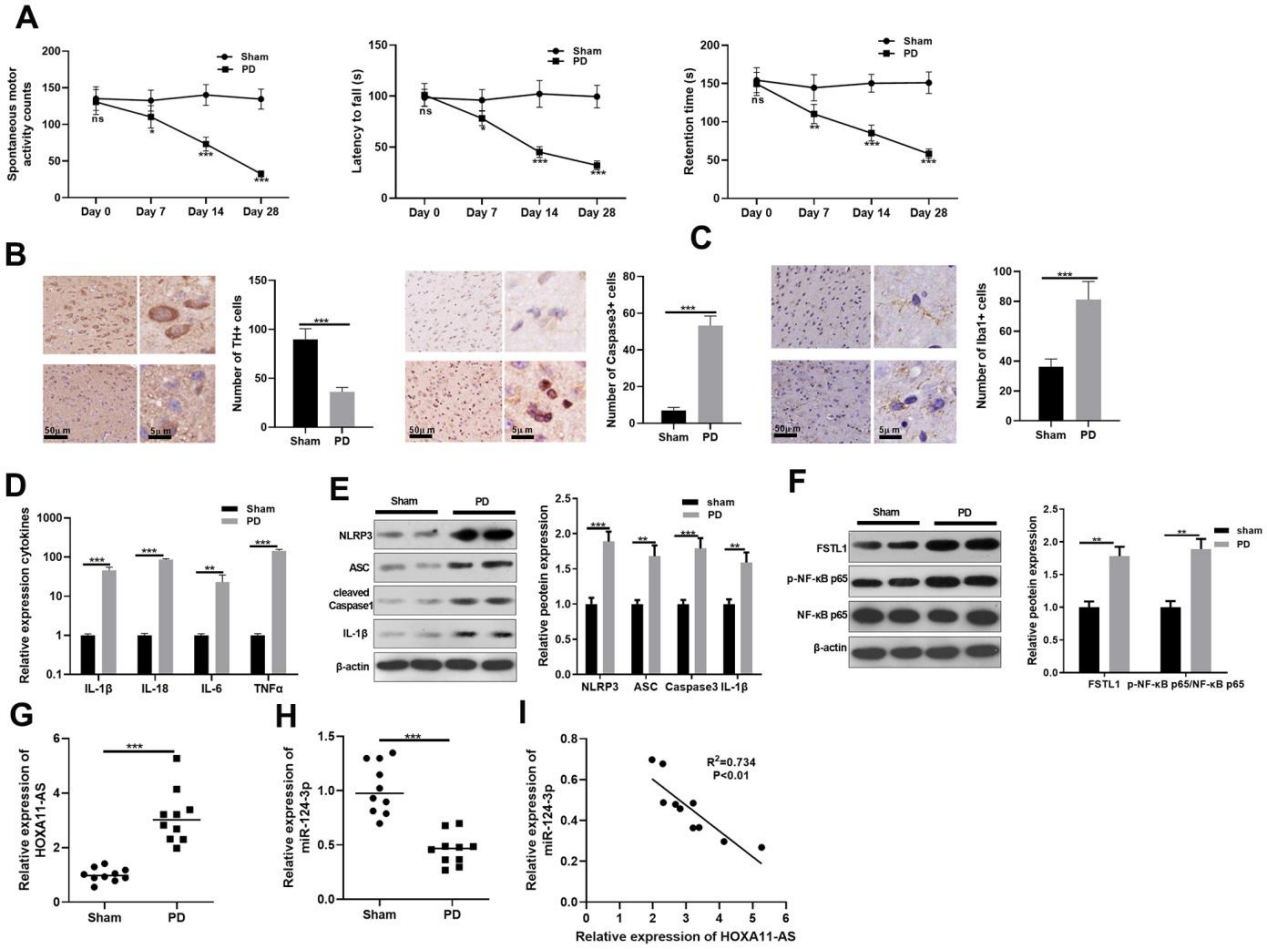
**Expression characteristics of LncRNA HOXA11-AS and miR-124-3p in PD model.** MPTP was used to induce a PD model in mice. (**A**) The neurological functions of PD mice were evaluated by spontaneous motor test, rotarod test and bevel test. (**B**, **C**) Immunohistochemistry was adopted to examine the number of TH and Caspase-3 positive cells and the microglia activation in SN area. (**D**) RT-PCR was conducted to determine the level of inflammatory factors IL-1β, IL-18, IL-6 and TNF-α in SN area. (**E**, **F**) Western blot was carried out to monitor the expression of FSTL1, NF-κB and NLRP3 inflammasome in SN area. (**G**, **H**) RT-PCR was adopted to examine the expression of HOXA11-AS and miR-124-3p in in SN area. (**I**) Person was applied to analyze the correlation between HOXA11-AS and miR-124-3p. ns *P*> 0.05, * *P* <0.05, ** *P* <0.01, *** *P* <0.001 (vs. sham group).

Firstly, we constructed a mouse PD model induced by MPTP to explore HOXA11-AS and miR-124-3p expressions. Then, spontaneous motor activity test, rotarod experiment and bevel test were conducted to evaluate the neurologic function of mice. Interestingly, compared with the sham group, the mice’ spontaneous motor activity was distinctly reduced, the time to fall off the rotard rod was shortened, and the time to stay on the bevel was decreased in PD mice, indicating that the PD mice had obvious behavioral disorders (*P* <0.05, Figure 1A). Afterward, immunohistochemistry was employed to calculate the number of TH and Caspase-3 positive cells in the substantia nigra (SN) area. It was found that compared with the sham group, the TH positive cell was reduced, while the Caspase-3 apoptotic cell was distinctly increased (*P* <0.05, Figure 1B), and the microglia was obviously activated (*P* <0.05, Figure 1C) in the PD group. Additionally, RT-PCR showed that the inflammatory factors IL-1β, IL-18, IL-6 and TNF-α were all up-regulated in the PD mouse (*P* <0.05, Figure 1D). Next, the level of FSTL1, NF-κB and NLRP3 inflammasome was detected by Western blot. The results illustrated that FSTL1, p-NF-κB p65, NLRP3, ASC, Caspase1 and IL-1β were all up-regulated compared with that of the sham group. (*P* <0.05, Figure 1E, 1F). Besides, we monitored the expression of HOXA11-AS and miR-124-3p with RT-PCR. As a result, HOXA11-AS was up-regulated in the PD model, while miR-124-3p expression was abated (*P* <0.05, Figure 1G, 1H). Further, the results of Person analysis demonstrated that the level of HOXA11-AS and miR-124-3p was negatively correlated (R^2^ = 0.734, *P* <0.01, Figure 1I). The results of above suggest that HOXA11-AS possibly participated in the regulating the PD model of mice via sponging miR-124-3p, which need further explore.

### The effect of HOXA11-AS and miR-124-3p on MPTP-mediated SH-SY5Y neuronal damage

**Figure 2:**
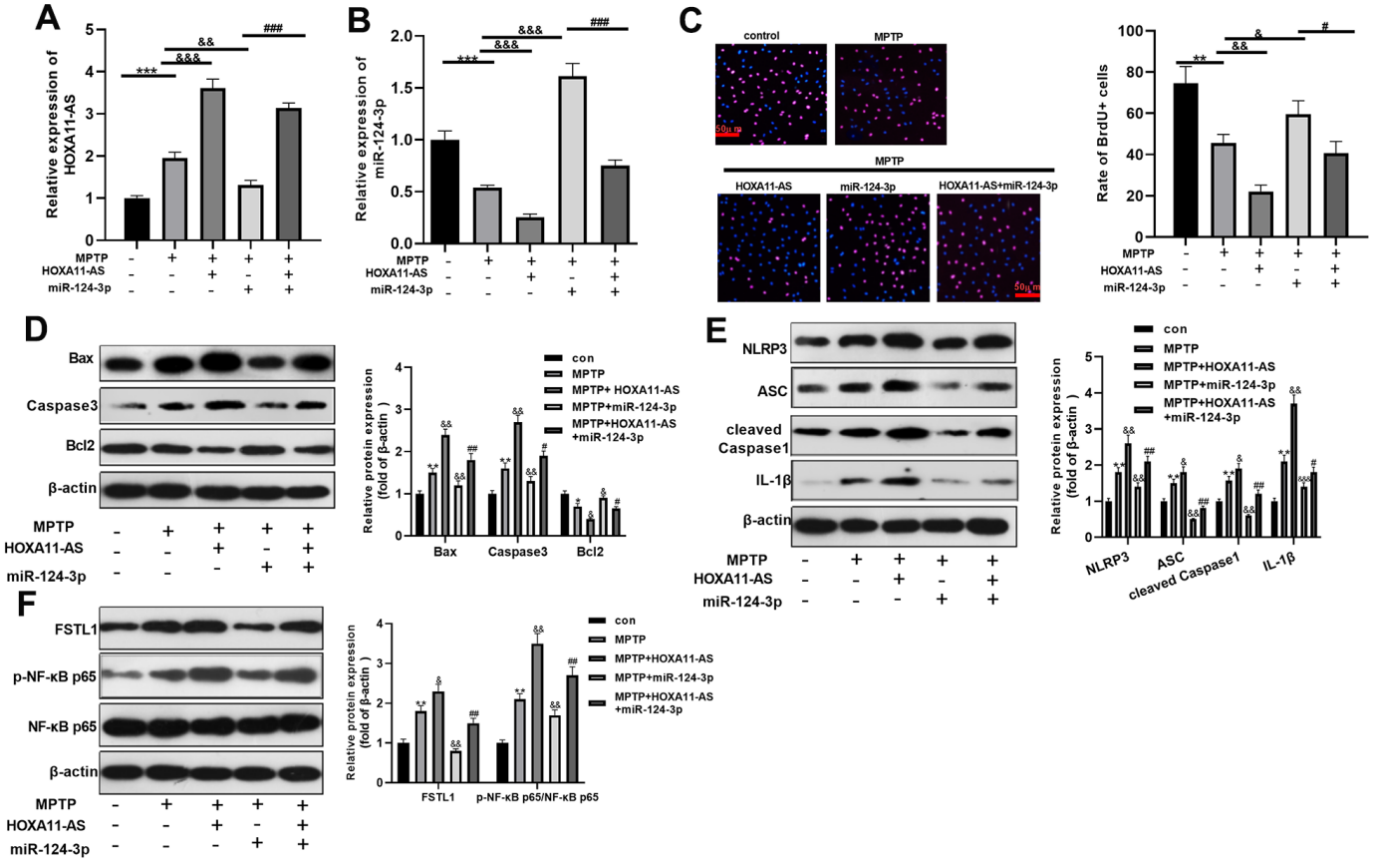
**The effect of HOXA11-AS and miR-124-3p on MPTP-mediated SH-SY5Y neuronal damage.** An *in vitro* model of PD was induced by MPTP on SH-SY5Y neuron cells. (**A**, **B**) RT-PCR was used to monitor HOXA11-AS and miR-124-3p expressions in SH-SY5Y cells. (**C**, **D**) Brdu and Western blot experiments were carried out to test cell viability and expression of apoptosis-related proteins in SH-SY5Y cells. (**E**, **F**) Western blot was applied to verify the protein expression of NLRP3 inflammasome, FSTL1 and p-NF-κB in SH-SY5Y cells. ** *P* <0.01, *** *P* <0.001 (vs.con group), & *P* <0.05, && *P* <0.01, &&& *P* <0.001 (vs.MPTP group), #*P* <0.05, ### *P* <0.001 (vs.MPTP + miR-124-3p group).

To explore the effect of HOXA11-AS and miR-124-3p on MPTP-mediated SH-SY5Y neurons, we treat SH-SY5Y with MPTP, and intervened with or without HOXA11-AS over-expressing plasmid and miR-124-3p mimic separately. RT-PCR was conducted to monitor HOXA11-AS and miR-124-3p expressions. Interestingly, compared with the MPTP group, HOXA11-AS expression was dramatically elevated after over-expressing HOXA11-AS, while miR-124-3p mimics notably down-regulated HOXA11-AS. Additionally, HOXA11-AS was obviously up-regulated with the combined intervention of the HOXA11-AS over-expressing plasmid and miR-124-3p mimic, when compared with that of miR-124-3p mimics alone. The same method was utilized to test miR-124-3p expression, and the complete opposite result was obtained (*P* <0.05, Figure 2A, 2B). Besides, BrdU and Western blot were conducted to study the cell viability and apoptosis. The results proved that compared with the MPTP group, the cell viability and Bcl2 expression were notably attenuated, and the pro-apoptotic proteins Bax and Caspase3 were obviously up-regulated after over-expressing HOXA11-AS. Additionally, miR-124-3p upregulation enhanced cell viability and inhibited apoptosis, which were significantly repressed by HOXA11-AS upregulation (*P* <0.05, Figure 2C, 2D). Further, Western blot revealed that compared with the MPTP group, the NLRP3, ASC, Caspase1, IL-1β, FSTL1 and p-NF-κB p65 were obviously up-regulated after over-expressing HOXA11-AS, while were markedly down-regulated after downregulating miR-124-3p. Furthermore, compared with MPTP+miR-124-3p group, enhancing HOXA11-AS level promoted the expression of the above proteins (*P* <0.05, Figure 2E, 2F). Collectively, HOXA11-AS aggravated MPTP-mediated SH-SY5Y damage through inhibiting miR-124-3p.

### The effect of HOXA11-AS and miR-124-3p on LPS-mediated BV2 microglia activation

**Figure 3:**
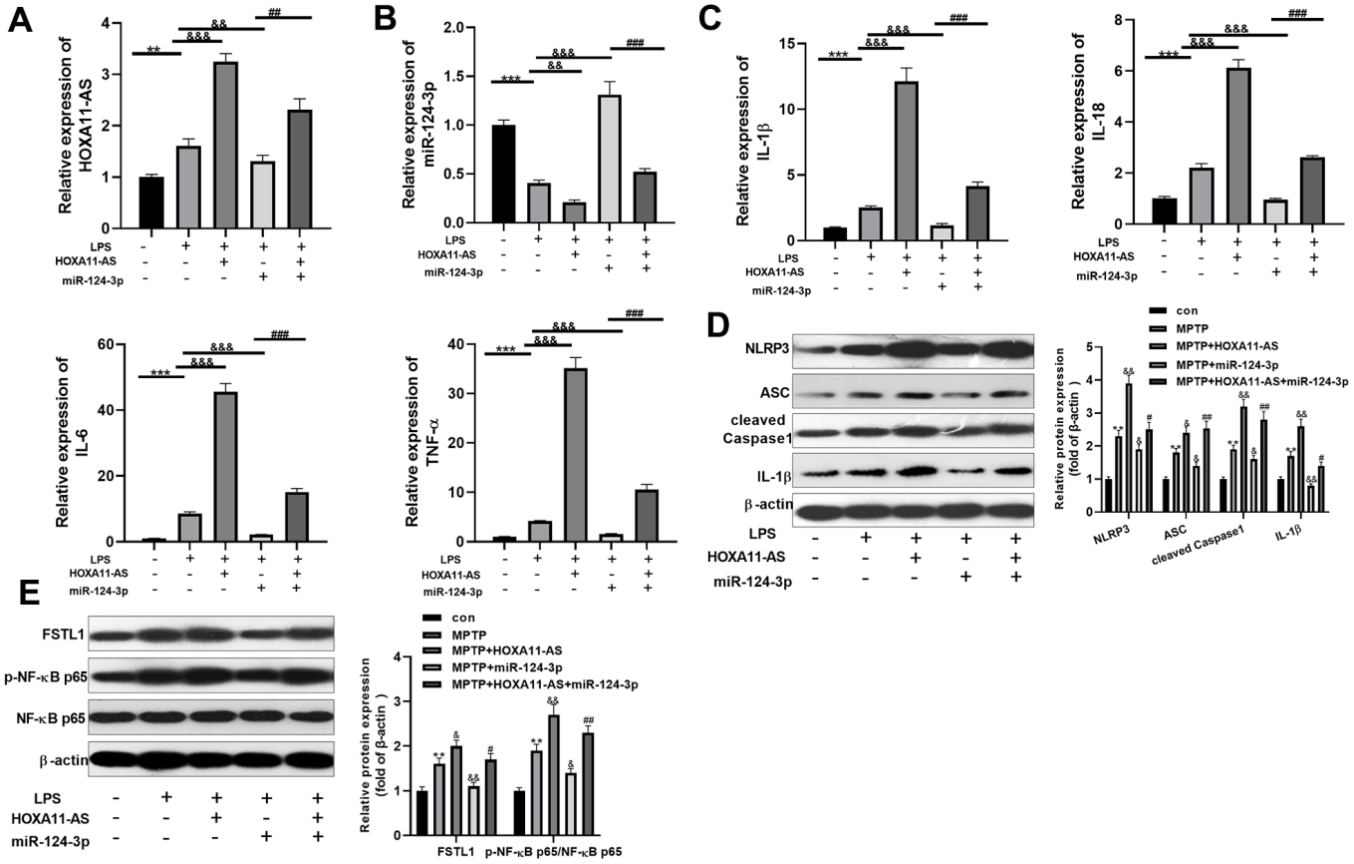
**Effect of HOXA11-AS and miR-124-3p on LPS-mediated activation of BV2.** An *in vitro* model of PD was induced by LPS on BV2 cells. (**A**, **B**) RT-PCR was employed to monitor HOXA11-AS and miR-124-3p expressions in BV2 microglia cells. (**C**) RT-PCR was carried out to test the level of inflammatory factors IL-1β, IL-18, IL-6 and TNF-α in BV2 microglia cells. (**D**, **E**) Western blot test was performed to determine the protein expression of NLRP3 inflammasome, FSTL1 and p-NF-κB in BV2 microglia cells. ** *P* <0.01, *** *P* <0.001 (vs.con group), && *P* <0.01, &&& *P* <0.001 (vs.LPS group), ### P <0.001 (vs. LPS + miR-124-3p group).

To probe into the function of HOXA11-AS and miR-124-3p on LPS-mediated BV2 cell, we administered the two on the basis of LPS-induced BV2 microglia. RT-PCR was conducted to test HOXA11-AS and miR-124-3p expressions. As a result, compared with the LPS group, HOXA11-AS was notably up-regulated after over-expressing HOXA11-AS, while it was distinctly down-regulated after miR-124-3p mimics transfection (*P* <0.05, Figure 3A, 3B). Besides, RT-PCR demonstrated that over-expressing HOXA11-AS facilitated the expression of inflammatory factors IL-1β, IL-18, IL-6 and TNF-α, which were attenuated by miR-124-3p mimics. Moreover, over-expressing HOXA11-AS mainly restrained miR-124-3p mediated effects (*P* <0.05, Figure 3C). Furthermore, the level of FSTL1, p-NF-κB p65 and NLRP3 inflammasome was compared by Western blot. The results illustrated that they were all up-regulated by over-expressing HOXA11-AS, while were dramatically attenuated by miR-124-3p mimics. However, over-expressing HOXA11-AS almost offset miR-124-3p-induced inhibition on the level of FSTL1, p-NF-κB p65 and NLRP3 inflammasome (*P* <0.05, Figure 3D, 3E). The above results further indicated that HOXA11-AS promoted BV2 microglia activation, while miR-124-3p could reverse the effect of HOXA11-AS.

### miR-124-3p was the target of HOXA11-AS and FSTL1

**Figure 4:**
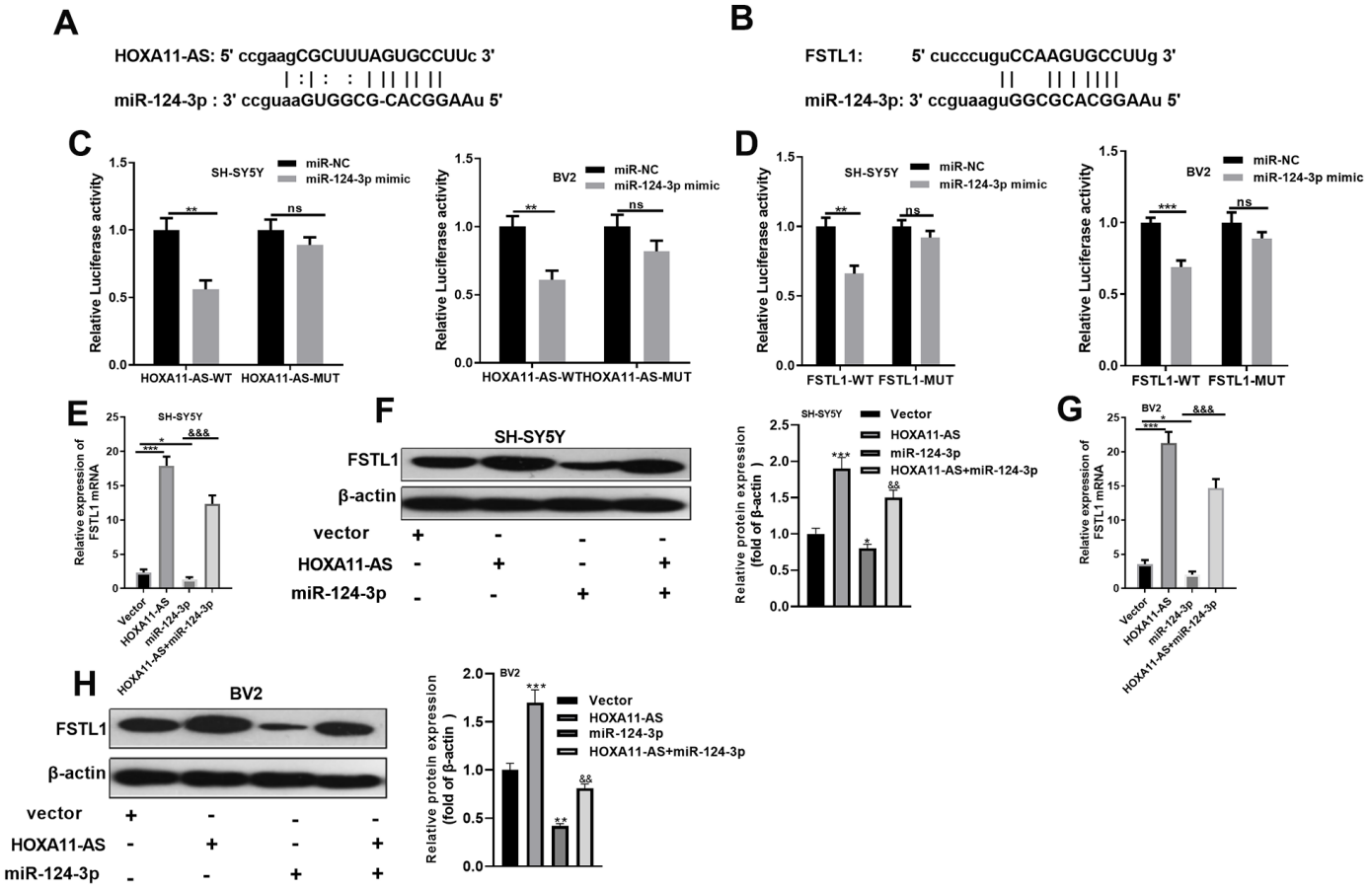
**miR-124-3p was the target of HOXA11-AS and FSTL1.** (**A**, **B**) The targeted association between HOXA11-AS, miR-124-3p and FSTL1 was found in the Starbase database (http://starbase.sysu.edu.cn/). (**C**, **D**) Dual-luciferase reporter assay was conducted to verify the targeting relationship between HOXA11-AS and miR-124-3p, miR-124-3p and FSTL1 in SH-SY5Y and BV2 cells. (**E**–**H**) RT-PCR and Western blot experiments were adopted to determine FSTL1 mRNA and protein expressions. ns*P*> 0.05, ** *P* <0.01, *** *P* <0.001 (vs.miR-NC group) *P <0.05, *** *P* <0.001 (vs.vestor group) &&& *P* <0.001 (vs.miR-124 -3p group).

In view of the lncRNA-miRNA-mRNA regulatory network, we were curious about the targeting association between HOXA11-AS, miR-124-3p, and FSTL1. Through the Starbase database (http://starbase.sysu.edu.cn/), HOXA11-AS was found to target miR-124-3p (Figure 4A). Also, there was a binding relationship between FSTL1 and miR-124-3p (Figure 4B). To verify the targeting relationship, dual-luciferase reporter assay was conducted in SH-SY5Y neurons and BV2 cells. The results proved that miR-124-3p obviously abated the luciferase activity of HOXA11-AS-WT and FSTL1-WT (*P* <0.05, Figure 4C, 4D), while had little effect on that of the HOXA11-AS-MUT and FSTL1-MUT (*P*> 0.05, Figure 4C, 4D). Further, RT-PCR and Western blot experiments turned out that, compared with the control group, the mRNA and protein expression of FSTL1 were both facilitated after HOXA11-AS alone intervention, while were dampened after adding miR-124-3p mimic alone. And the joint intervention of HOXA11-AS and miR-124-3p mimic dramatically reversed the down-regulation effect of miR-124-3p mimic only (*P* <0.05, Figure 4E, 4F). These findings illustrated that there was a binding association among HOXA11-AS, miR-124-3p and FSTL1. MiR-124-3p was an essential target of HOXA11-AS and FSTL1.

### Inhibition of NF-κB attenuated HOXA11-AS-mediated neuronal damage and microglia activation

**Figure 5:**
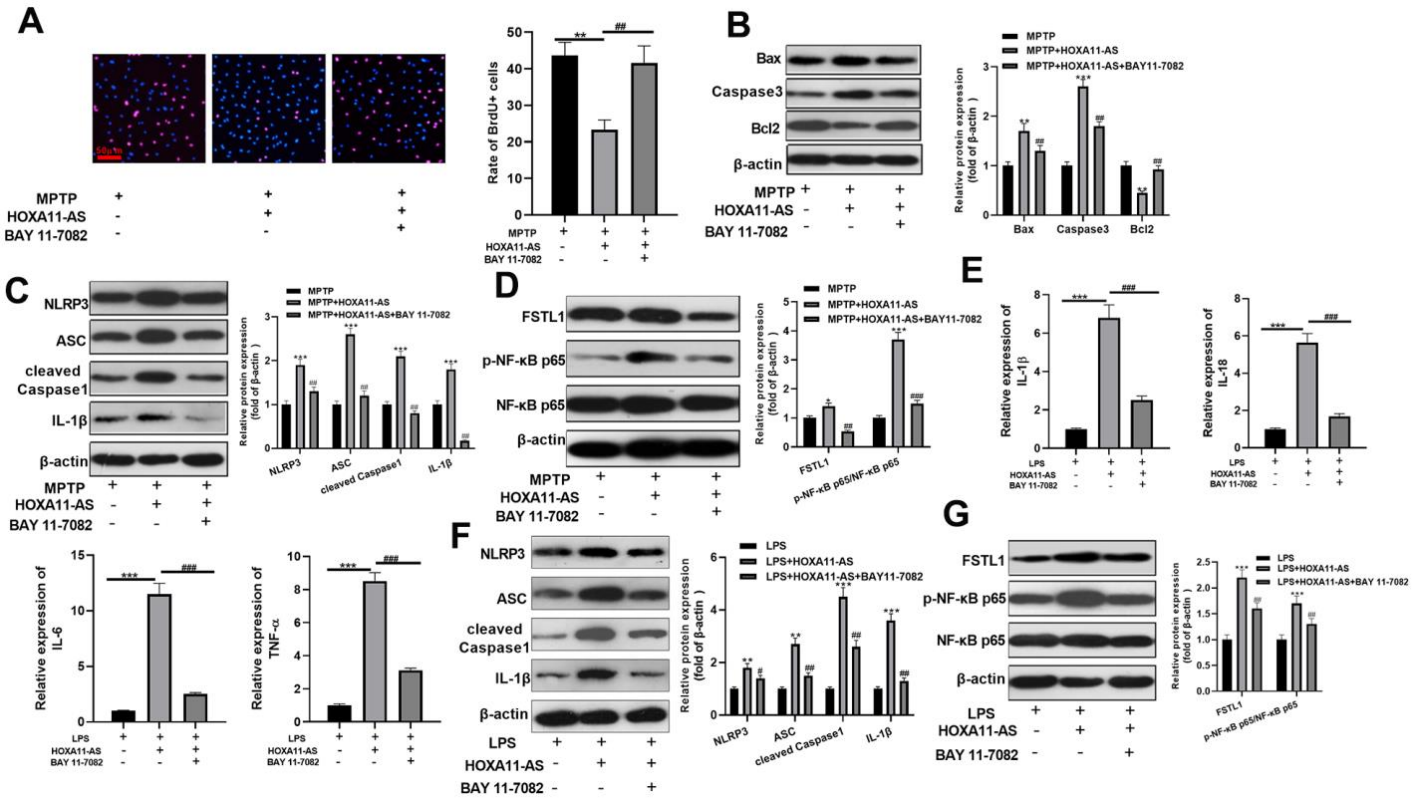
**Inhibition of NF-κB attenuated HOXA11-AS-mediated neuronal damage and microglia activation.** MPTP induced SH-SY5Y cells and LPS induced BV2 cells were transfected HOXA11-AS overexpressing and/or treated with BAY 11-7082 (1 μmol/L). (**A**) Brdu assay was conducted to test the cell viability of SH-SY5Y cells. (**B**–**D**) Western blot was utilized to compare the expression of apoptosis-related proteins (Bax, Caspase3 and Bcl2), NLRP3 inflammasome, FSTL1 and NF-κB in SH-SY5Y cells. (**E**) RT-PCR was carried out to test the level of inflammatory factors IL-1β, IL-18, IL-6 and TNF-α in BV2 cells. (**F**, **G**) Western blot was employed to monitor the protein expression of NLRP3 inflammasome, FSTL1 and NF-κB in BV2 cells. ** *P* <0.01, *** *P* <0.001 (vs.MPTP or LPS group), ## *P* <0.01, ### *P* <0.001 (vs.MPTP + HXOA11-AS or LPS+HOXA11-AS group).

To investigate the function of NF-κB pathway in HOXA11-AS-mediated neuronal damage and microglial inflammation, we administered the NF-κB inhibitor BAY 11-7082 into the cells. The results of BrdU assay showed that the cell viability was dampened after over-expressing HOXA11-AS, while it was obviously strengthened after inhibition p-NF-κB p65 via giving BAY 11-7082 (*P* <0.05, Figure 5A). Meanwhile, western blot results found that over-expressing HOXA11-AS up-regulated the pro-apoptotic proteins Bax and Caspase3, and NLRP3 inflammasome, FSTL1, and p-NF-κB p65, while down-regulated the anti-apoptotic protein Bcl2. Moreover, BAY 11-7082 intervention on this basis notably reversed the HOXA11-AS-mediated neuronal injury (*P*<0.05, Figure 5B–5D). The experiments in microglia also confirmed that over-expressing HOXA11-AS elevated the level of inflammatory factors IL-1β, IL-18, IL-6 and TNF-α, and the effects were weakened by BAY 11-7082 (*P* <0.05, Figure 5E). Furthermore, results of western blot reveled that over-expressing HOXA11-AS increased pro-apoptotic proteins, NLRP3 inflammasome and p-NF-κB p65 expressions, while supplementing NF-κB inhibitor distinctly dampened the effect of HOXA11-AS alone intervention (*P* <0.05, Figure 5F, 5G). Above results demonstrated that inhibiting NF-κB alleviated HOXA11-AS-mediated neuronal damage and microglia activation.

### Knocking down HOXA11-AS mitigated PD progression and inflammation in mice

**Figure 6:**
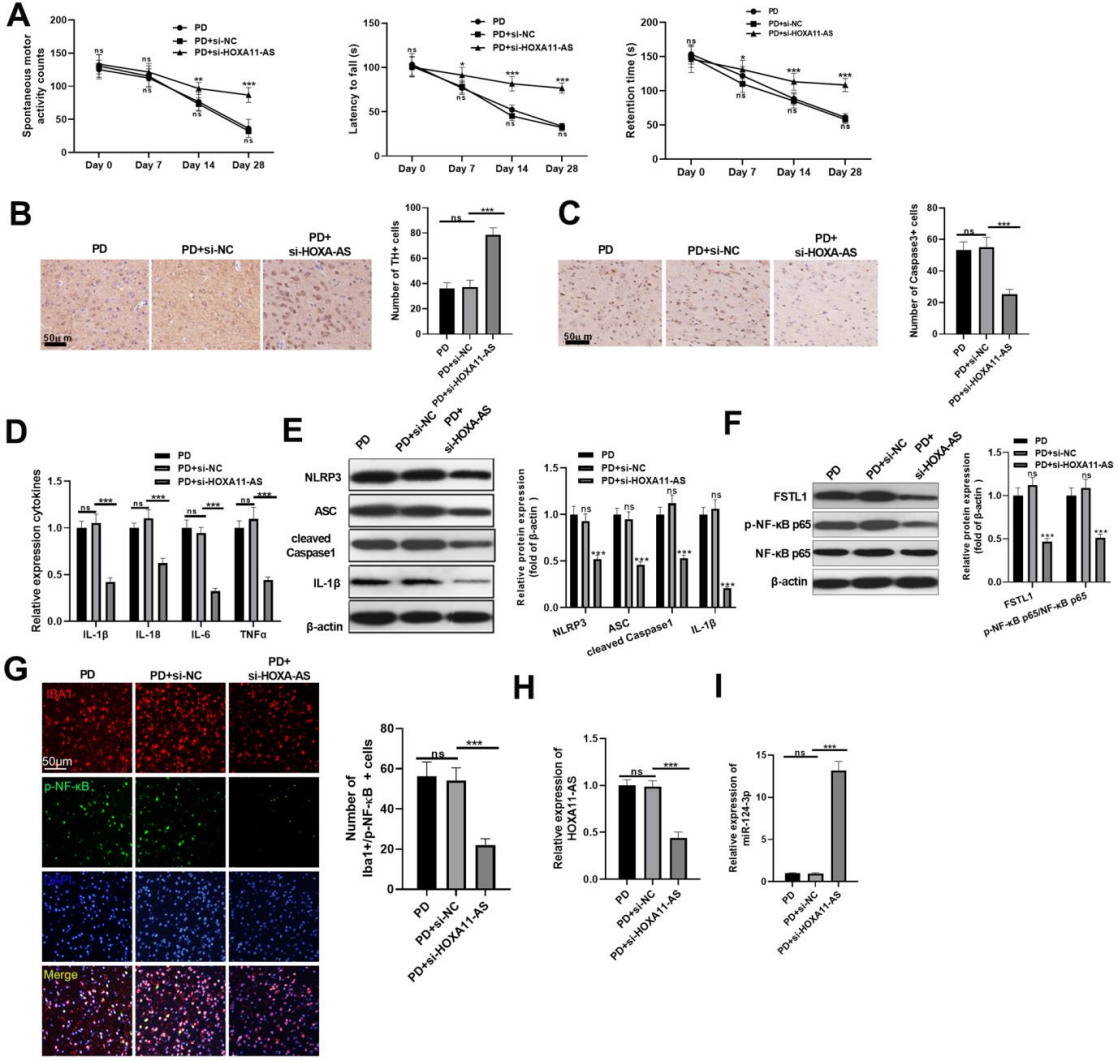
**Knocking down HOXA11-AS mitigated PD progression and inflammation in mice.** Si-HOXA11-AS was used to inference the level of HOXA11-AS in the brain tissues of mice, which were then subjected to MPTP to induce a PD model. (**A**) The neurological functions of PD mice were evaluated by spontaneous motor test, rotarod test and bevel test. (**B**, **C**) Immunohistochemistry was adopted to examine the number of TH (**B**) and Caspase-3 (**C**) positive cells in SN area. (**D**) RT-PCR was conducted to determine the level of inflammatory factors IL-1β, IL-18, IL-6 and TNF-α in SN area. (**E**, **F**) Western blot was carried out to monitor the expression of FSTL1, NF-κB and NLRP3 inflammasome in SN area. (**G**) The activation of microglia was tested by immunofluorescence. (**H**, **I**) RT-PCR was adopted to examine the expression of HOXA11-AS and miR-124-3p in in SN area. ns *P*> 0.05 (vs.PD group), ns *P*> 0.05, * *P* <0.05, ** *P* <0.01, *** *P* <0.001 (vs.PD + Si-NC group).

We constructed a PD model *in vivo* to further explore the effects of HOXA11-AS in PD development. The neurological functions of mice were evaluated by the spontaneous motor activity test, rotarod experiment and bevel test. It was discovered that compared with the PD+si-NC group, the spontaneous motor activity was increased, the time to fall off the rotard rod was extended, and the time staying on the bevel was increased after downregulating HOXA11-AS, indicating that repressing HOXA11-AS1 mitigated neurological deficits of PD mice (*P* <0.05, Figure 6A). Besides, downregulating HOXA11-AS also increased TH-labeled neurons and attenuated Caspase-3 positive cells in the SN area (*P<* 0.05, Figure 6B, 6C). Moreover, RT-PCR showed that the inflammatory factors IL-1β, IL-18, IL-6 and TNF-α were all dramatically attenuated after knocking down HOXA11-AS (compared with PD+si-NC group) (*P*<0.05, Figure 6D). Next, the level of FSTL1, NF-κB and NLRP3 inflammasome were examined by Western blot. It turned out that knocking down HOXA11-AS markedly inhibited the above proteins (Figure 6E, 6F). Further, the results of immunofluorescence confirmed that the microglia activation was weakened, and the number of p-NF-κB p65 was reduced after knocking down HOXA11-AS (*P* <0.05, Figure 6G). Furthermore, the results of RT-PCR showed that HOXA11-AS was down-regulated, while miR-124-3p was up-regulated in the PD+si-HOXA11-AS group (compared with PD+si-NC group) (*P* <0.05, Figure 6H, 6I), proving that knocking down HOXA11-AS mitigated the PD devolvement and inflammation in mice.

## DISCUSSION

PD is a kind of neurodegenerative disease with selective degeneration of dopaminergic neurons in the substantia nigra and the presence of Lewy bodies in the remaining neurons. LncRNA and miR has been found play vital role in PD development [24–26]. So, *in vivo* and *in vitro* experiments, we proved that inhibition of HOXA11-AS alleviating the neuron damage and microglial neuroinflammation via regulating miR-124-3p-FSTL1-NF-κB axis.

Recently, lncRNAs have been identified as powerful regulators in CNS physiological processes and diseases [27, 28]. For example, HOXA11-AS is dysregulated in mangy cancers and contributes to tumor development as whether a tumor inhibitor or an oncogene [29–33]. Studies have shown that HOXA11-AS is a dominant regulator in inflammation. Li Y et al. confirmed that HOXA11-AS mediates calcium oxalate (CaOx) kidney stones-induced ephritis, and modulates MCP-1 expression by targeting miR-124-3p, thus enhancing apoptosis and exacerbating cell damage in HK-2 [13]. This study firstly confirmed that HOXA11-AS is notably over-expressed in the PD mice and LPS-induced microglia. Additionally, HOXA11-AS increases the level of inflammatory factors IL-1β, IL-18, IL-6 and TNF-α, and activates microglia, while inhibiting HOXA11-AS distinctly alleviates MPTP-mediated neuronal damage and microglia activation no matter *in vivo* and *in vitro*. The above results showed that HOXA11-AS is involved in the PD development as one neuroprotective and anti-inflammatory gene.

miRNAs have been reported to exert a critical function in the pathogenesis of PD [34]. Also, miR-124-3p is an important non-coding RNA. One study found that miR-124-3p play a protective role in PD, which enhances 6-hydroxydopamine (6-OHDA)-induced PC12 and SH-SY5Y cell viability by targeting ANXA5 [35]. Besides, research by Geng L et al. revealed that miR-124-3p is down-regulated in methyl phenyl pyridinium iodide(MPP)-induced SH-SY5Y cells, which elevates cell viability and superoxide dismutase activity, and reduces the inflammatory factor TNF-α and IL-1β by targeting STAT3, thereby relieving neuron damage [36]. Similar to the above results, our study also illustrated that miR-124-3p was down-regulated in both PD mice and LPS-induced microglia, and over-expressing miR-124-3p attenuated MPTP-mediated neuronal damage and microglia activation.

Studies have shown that FSTL1 is involved in various inflammation-related diseases [37]. Recent studies have found that FSTL1 exerts a vital role in inflammations by activating the NF-κB pathway. For example, knocking down FSTL1 protects oxidized low-density lipoprotein (OxLDL)-induced human coronary artery vascular cell (HCAECs) injury models by inhibiting TLR4/MyD88/NF-κB and MAPK signaling pathways [21]. In addition, Qu Y et al. found that the NF-κB signaling pathway is activated in both degenerated cartilage and FSTL1-treated chondrocytes. Meanwhile, intervention of NF-κB inhibitor dramatically dampens FSTL1-induced inflammatory cytokines and proteolytic enzyme over-expression [20]. Meanwhile, there are also reports that FSTL1 downregulates pro-inflammatory cytokines by inhibiting the MAPK/p-ERK1/2 pathway in astrocytes, thus exerting an anti-inflammatory role in CNS inflammation [38]. These results suggest that FSTL1 in inflammations is controversial, and FSTL1 is expressed differently in diversified cell types. Our study affirmed that there was a binding association between FSTL1 and miR-124-3p. Over-expressing HOXA11-AS obviously strengthened FSTL1 expression, while miR-124-3p down-regulated FSTL1. Meanwhile, FSTL1 and NF-κB were both up-regulated in MPTP-mediated SH-SY5Y neuronal injury and LPS-mediated microglia activation. Further, BAY 11-7082 was applied to inhibit NF-κB expression. As a result, BAY 11-7082 obviously reversed HOXA11-AS-mediated neuronal damage and microglia activation, suggesting that HOXA11-AS accelerated the PD inflammation through the FSTL1-NF-κB axis.

**Figure 7:**
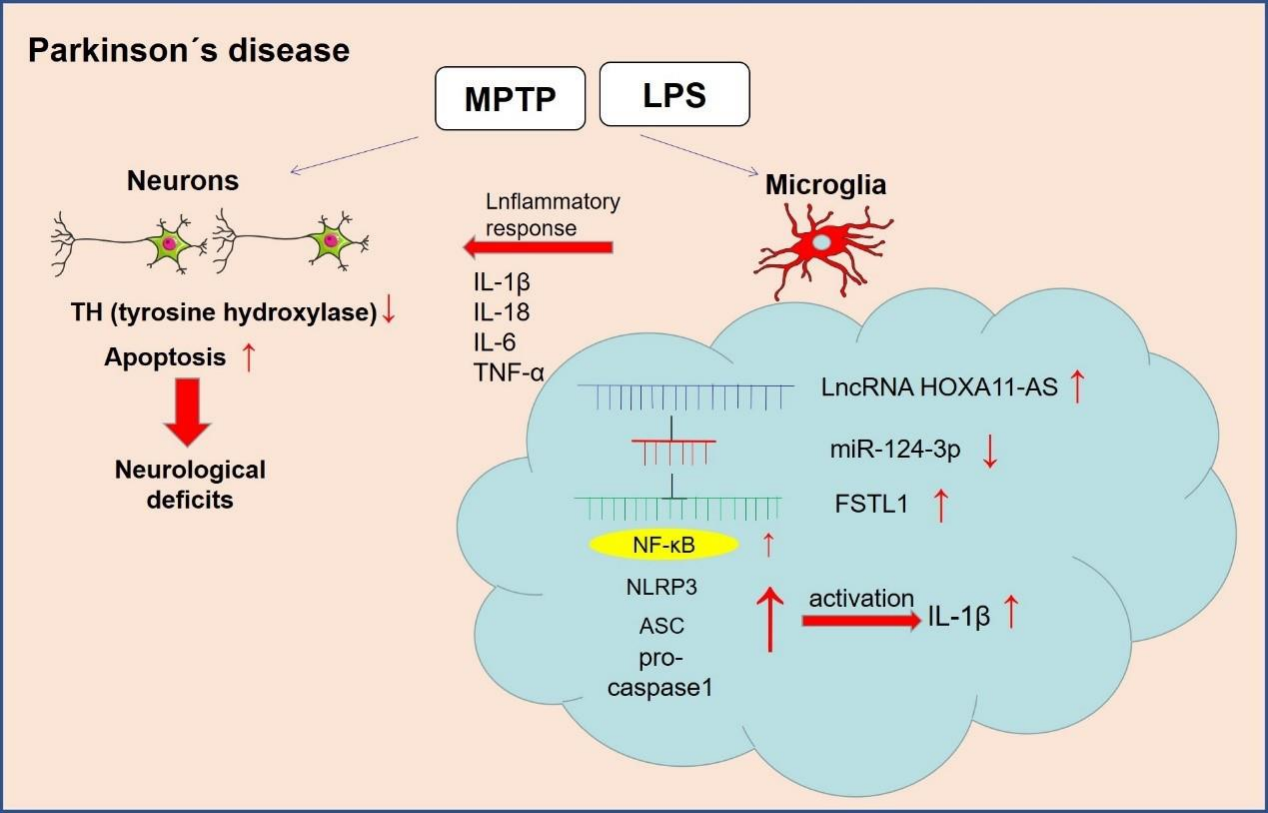
**The mechanism diagram of the present study.** LncRNA HOXA11-AS was up-regulated in the PD model. Inhibition of HOXA11-AS protects mice against PD through repressing neuroinflammation and neuronal apoptosis through miR-124-3p-FSTL1-NF-κB axis.

NLRP3 inflammasome belongs to the NLR family, which activates the cleavage of Caspase-1, IL-1β and IL-18 precursors, leading to the inflammatory reactions [39]. Related researches manifest that NLRP3-mediated neuroinflammation is closely associated with PD evolvement. Studies have shown that α-synuclein mediates the activation of NLRP3 inflammasome by up-regulating Atg5 to promote PD progression [40]. Besides, Sun Q et al. found that miR-190 negatively regulates the expression and activation of NLRP3 inflammasome in the MPTP-induced PD mouse model to reduce neuronal damage and inhibit inflammation [41]. Here, western blot revealed that over-expressing HOXA11-AS promoted the expression of FSTL1, NF-κB and NLRP3 inflammasome, while the administration of miR-124-3p mimics reversed the HOXA11-AS-induced up-regulation of the above proteins. It was demonstrated that HOXA11-AS facilitated the expression of inflammatory factors and microglia activation by regulating the miR-124-3p-FSTL1/NF-κB axis (Figure 7).

Collectively, we confirmed that HOXA11-AS was up-regulated in PD, and promoted the release of inflammatory factors by targeting the miR-124-3p-NF-κB axis. Meanwhile, inhibiting HOXA11-AS notably increased miR-124-3p expression to attenuate the FSTL1/NF-κB axis, thus dampening the neuron damage, expression of inflammatory factors and NLRP3 inflammasome, thereby alleviating PD progression. This study provides an important reference for the diagnosis and treatment of PD, while more related mechanisms and treatment strategies need further exploration.

## MATERIALS AND METHODS

### Experimental animals

C57BL/6J male mice aged 8-10 weeks were purchased from Nanjing Laboratory Animal Center (Nanjing, China), and kept in sterile equipment at 25 ± 2° C, with 50–60% relative humidity in a 24 hour of light/darkness environment, having free access to water and food. The mice were randomly divided into sham group and PD group. The PD model was constructed by intraperitoneal injection of MPTP (Sigma, USA) (20 mg/kg, 3 times/day, 8-hour interval) for 4 consecutive weeks. The mice in the sham group were injected with the same amount of normal saline. Behavioral tests were performed on days 0, 7, 14, and 28. After behavioral assessment on day 28, mice in the control group and PD group were anesthetized and perfused, and immunohistochemical assessment was performed on TH labeled dopaminergic neurons. Twenty-four hours after the last injection of MPTP, the mice were decapitated, and the midbrain tissue was taken and stored at -8° C for subsequent experiments. To evaluate the effect of HOXA11-AS on MPTP-induced PD, the small inference RNA targeting HOXA11-AS (si-HOXA11-AS) and the siRNA-negative control (si-NC) were respectively injected into the midbrain of PD mice 2 days before the administration of MPTP into the mice. All surgical operations were performed under anesthesia and approved by the Animal Ethics Committee of Kaifeng central hospital neurology. The experiment was conducted strictly following the Laboratory Animal Care and Use Guidelines of the National Institutes of Health (NIH Publication No. 8023).

### Behavioural assessments of mice

1) Spontaneous motor experiment: The spontaneous motor activity was monitored with a computerized motion detection system with infrared sensors (manufactured by the Institute of Materia Medica of the Chinese Academy of Medical Sciences and Beijing Union Medical College). Before the test, the mice were put in the transparent plexiglass cylinder with a diameter of 40 cm and a height of 13 cm to make them adapt to the environment for 5 min. Then, the number of horizontal and vertical motions were recorded within 10 min, and the result was expressed as a cumulative count within 10 min. 2) Rotarod test: Firstly, the mice were fixed on a fixed rod with a diameter of 3 cm for 30 s, and any fallen mice were put back on the fixed rod during this period. Next, the mice received constant speed training at 15 r/min for 120 s. The mice that failed the first adjustment were given two additional adjustments. Thirty minutes after the last training session, the mice were placed on a pole, timed to determine their motor skills. Namely, the mice received a constant speed of 15 r/min with the time limit of 120 s and the interval of 30 min, and the training was repeated three times. 3) Bevel experiment: the mice were placed on the rough bevel at a 60° C angle for the bevel test. The time each mouse stayed on the bevel was recorded. If the mice maintained on the bevel for more than 3 min, the record was 180 s, and each animal was tested in triplicate.

### Cell culture and treatment

Dopaminergic neuron cells SH-SY5Y and BV-2 microglia were purchased from the Chinese Academy of Sciences (Shanghai, China), and then cultured in DMEM medium (Thermo Fisher HyClone, Utah, USA) containing 5% FBS (Thermo Fisher Scientific, MA, USA) in an incubator with 5% CO_2_ at 37° C. The medium was altered every 2 days, and the cells were sub-cultured once every 5 days. The experiment was performed when the cells covered about 90% of the bottom of the bottle. The cultured cells were harvested and inoculated in 6-well plates at about 5×10^6^/well, and incubated for 24 hours until the cells were in good condition. The *in vitro* PD model on SH-SY5Y cells was induced by MPTP (20 μg/mL), and on microglia was mediated by 1μg/mL LPS. Both LPS and MPTP were purchased from Sigma (Shanghai, China). The NF-κB inhibitor BAY 11-7082 (MedChemExpress: HY-13453, Shanghai, China) was used to treat SH-SY5Y and BV-2 cells at 1 μmol/L.

### Cell transfection

Cells in logarithmic growth phase were taken, and then seeded in 6-well plates at 5×10^6^/well after trypsinization. The cells were transfected after the cell growth was stable. HOXA11-AS over-expression plasmid (HOXA11-AS), si-HOXA11-AS, miR-124-3p mimics and their negative controls were obtained from GenePharma (Shanghai, China). The cells were incubated at 37° C with 5% CO_2_, and were transfected with Lipofectamine® 3000 (Invitrogen; ThermoFisher Scientific, Inc.) reagent according to the instructions of FuGENE® HD Transfection Reagent (Roche, Shanghai, China). After 24 hours of transfection, total cellular RNA was extracted for real-time fluorescence quantitative PCR to monitor the changes in miR-124-3p and HOXA11-AS expressions in the transfected cells.

### BrdU assay

SH-SY5Y cells in the logarithmic growth phase were taken to make a single-cell suspension, which was then inoculated in 24-well plates at 1×10^5^/well. After the cells were adherent to the wall, BrdU (AmyJet Scientific Inc. Wuhan, China) was added according to the operating instructions, and the plates were cultured in an incubator with 5% CO_2_ at 37° C. After being continuously cultured for a total of 48 hours, the cells were then subjected to immunofluorescence staining. The BrdU positive cell number and the total DAPI positive cell number under 3 fields were randomly chosen and counted under a microscope. Cell proliferation rate= BrdU-positive cell number/DAPI-positive cell number, and the average value of the cell proliferation rate in the three visual fields was taken as the cell proliferation rate.

### Real-time polymerase chain reaction (RT-PCR)

TRIzol reagent (Invitrogen, Waltham, MA, USA) was utilized to extract total RNA from tissues or cells, and RNA concentration and purity was determined with Nanodrop-spectrophotometer. Afterward, 1 μg of total RNA was reverse-transcribed to synthesize its complementary DNA (cDNA) with the PrimeScript-RT Kit (Madison, WI, USA) following the manufacturer's protocol. Subsequently, SYBR® Premix-Ex-Taq ™ (Takara, TX, USA) and ABI7300 systems were adopted for RT-PCR of cDNA. The total volume of the PCR system was 30 μl, and each sample contained 300 ng cDNA. The amplification procedure was initial denaturation at 95° C for 10 min, and then 45 cycles were performed, namely 95° C for 10 s, 60° C for 30 s, and 85° C for 20 s. All fluorescence data were converted into relative quantification, U6 served as the internal reference of miR-124-3p, while β-actin served as that of the remaining detected molecules. RT-PCR reactions were performed three times. The primers were designed and synthesized by Guangzhou Ribo Biotechnology Co., Ltd., China. Primer sequences were shown in Table 1.

**Table 1:** Primer sequences of RT-PCR.

**Gene**	**Primer sequence(5`→3`)**
miR-124-3p	forward: AAGTACTCTAAGGCACGCGGT reverse:CAGTGCAGGGTCCGAGGT
IL-6	forward:ATGAACTCCTTCTCCACAAGCGC reverse:GAAGAGCCCTCAGGCTGGACTG
IL-1β	forward:TCCCTTCATCTTTGAAGAAGA reverse:GAGGCCCCAAGGCCACAGG
TNF-α	forward:CATCCTTTCTCCACTGCACG reverse:AGCCCTACTCTTTGCATGGT
IL-18	forward:GCTCACCACAACCTCTACCT reverse:TTCAAGACCAGCCTGACCAA
FSTL1	forward:CCCCTCTGTTTTGGTTGCTC reverse:CTTCACCATGGCAGACGATG
HOXA11-AS	forward:GATGTCAGCGCCTCTAAAGC reverse:ATCCCACCTTCTGTCCTTGG
β-actin	forward:TGTCACCAACTGGGACGATA reverse:GGGGTGTTGAAGGTCTCAAA
U6	forward: TGCGGGTGCTCGCTTCGGCAGC reverse:CCAGTGCAGG GTCCGAGGT

### Immunohistochemistry

The brain tissues were routinely paraffin-embedded and continuously sectioned into coronary sections with about 5 μm thick for immunohistochemical staining. Afterward, the sections were routinely dewaxed with xylene and hydrated with gradient alcohol. After the sections were blocked with 3% H_2_O_2_ for 10 min, the endogenous peroxidase was inactivated, and 0.01mol/L sodium citrate buffer was used for microwave repair (pH = 6.0, 15min). After blocking with 5% bovine serum albumin (BSA) for 20 min, the primary antibodies anti-Caspase3 (ab13847, 1: 100, Abcam, MA, USA) or anti-Iba1(ab178846, 1: 100, Abcam, MA, USA) was added and incubated with the sections overnight at 4° C. On the next day, the Goat Anti-Rabbit IgG H&L (HRP) (ab6721, 1: 100, Abcam, MA, USA) was added and incubated with the sections at room temperature for 60 min, and then developed with DAB after being washed with PBS. After hematoxylin counterstaining, the sections were dehydrated and transparentized, and mounted for microscopic examination. Image-Pro Plus image analysis software (MediaCybemetics, MD, USA) was employed to analyze the number of TH and Caspase3 positive cells and the microglia activation.

### Immunofluorescence

The frozen sections were taken out, hydrated with 0.01mol/L PBS for 20 min and repaired with citric acid in a microwave for about 5 min. Then, 0.3% Triton X-100 was employed for rupture of membranes for 30 min, and the sections were rinsed with 0.01mol/L PBS for 5min (× 3 times). Afterward, the sections were blocked with antigen blocking buffer for 1 hour, and the primary antibodies Anti-Iba1 antibody (ab15690, 1: 200, abcam) and anti-p-NF-κB antibody (ab86299, 1: 200, abcam) were added and incubated with the sections overnight at 4° C. After rinsing with PBS, the secondary antibodies Goat Anti-Mouse IgG H&L (Alexa Fluor® 647) (ab150115, 1: 200, abcam) and Donkey Anti-Rabbit IgG H&L (Alexa Fluor® 488) (ab150073, 1: 200, abcam) were added and incubated at 37° C in the dark for 1 hour. Subsequently, the sections were rinsed with PBS and blocked with mounting medium containing 4 '6-diamidine 2-phenylindole (DAPI) (purchased from Shanghai Beyotime Biotechnology Co., Ltd., China), and then observed under a fluorescence microscope. Three sections of roughly the same level of brain tissues were taken from each mouse to observe p-NF-κB and Iba1 positive microglia cells, and five fields (×200) were randomly chosen to calculate the positive cell number.

### Western blot

The brain tissues, SH-SY5Y neurons and BV2 cells were harvested and then washed with cold PBS 3 times. Then, 100~200 μL RIPA lysate (Beyotime Biotcchnology, Shanghai, China) was added to lyse the cells in ice water, and Bradford method was adopted to determine the protein concentration. Afterward, an equal volume of protein from each group was loaded on 10% SDS-PAGE electrophoresis, and the protein on the gel was transferred to PVDF membranes (Millipore, Bedford, MA, USA). Subsequently, the membranes were blocked at 4° C for 1 hour, and the primary antibody (concentration 1: 1000) Anti-FSTL1 antibody (ab11805), Anti-β-actin antibody (ab115777), Anti-p-NF-κB antibody (ab86299), Anti-NF-κB antibody (ab16502), Anti-NLRP3 antibody (ab214185), Anti-ASC antibody (ab180799), Anti-Caspase-1 antibody (ab74279), Anti-IL-1β antibody (ab7632), Anti-Caspase3 antibody (ab13847), Anti-Bcl2 antibody (ab182858) and Anti-Bax antibody (ab32503) were added for incubation overnight at 4° C. After that, the membranes were washed twice with TBST at room temperature, and incubated with fluorescein-labeled secondary antibody goat anti-rabbit (ab205718, 1: 2500) for 1 hour at room temperature. The above antibodies were obtained from Abcam, MA, USA. After being washed 3 times, the membranes were exposed with ECL developer (Millipore, Bedford, MA, USA) and imaged with a membrane scanner.

### Dual-luciferase reporter assay

A dual-luciferase reporter assay system (Promega, Madison, WI, USA) was applied to carry out the dual-luciferase reporter assay. Then, the target fragments of wild-type HOXA11-AS and FSTL1, mutant HOXA11-AS and FSTL1 were constructed and integrated into pGL3 vector (Promega, Madison, WI, USA) to form pGL3-HOXA11-AS-wild type (HOXA11-AS-WT), PGL3-HOXA11-AS-mutant (HOXA11-AS-MUT), pGL3-FSTL1-wild type (FSTL1-WT) and pGL3-mutant (FSTL1-MUT) reporter vector. Subsequently, HOXA11-AS/FSTL1-WT or HOXA11-AS/FSTL1-MUT was co-transfected with miR-124-3p or negative control into SH-SY5Y neuron and BV2 cells. Forty-eight hours later, luciferase activity was measured according to the manufacturer's instructions. All experiments were conducted in triplicate.

### Data analysis

The data in this study were all analyzed with SPSS22.0 statistical software (SPSS Inc., Chicago, IL, USA). Measurement data conforming to normal distribution were expressed as mean±standard deviation (x±s). One-way ANOVA was used for data comparison between groups, *t*-test was adopted for comparing measurement data between two groups, while person linear correlation analysis was applied for regression analysis. *P* <0.05 was considered statistically significant.
